# Training, experience, and perceptions of chest tube insertion by higher speciality trainees: implications for training, patient safety, and service delivery

**DOI:** 10.1186/s12909-023-04978-8

**Published:** 2024-01-03

**Authors:** Ben Probyn, Cyrus Daneshvar, Tristan Price

**Affiliations:** 1 University Hospitals Plymouth NHS Trust, Plymouth, England; 2 University of Plymouth, Plymouth, England

**Keywords:** Training, Physician, Seldinger chest tube insertion

## Abstract

**Background:**

Seldinger Chest Tube Insertion (CTI) is a high acuity low occurrence procedure and remains a core capability for UK physician higher speciality trainee’s (HST). A multitude of factors have emerged which may affect the opportunity of generalists to perform CTI. In view of which, this paper sought to establish the current experiences, attitudes, training, and knowledge of medical HST performing Seldinger CTI in acute care hospitals in the Peninsula deanery.

**Methods:**

A Scoping review was performed to establish the UK medical HST experience of adult seldinger CTI. Synonymous terms for CTI training were searched across Cochrane, ERIC, Pubmed and British education index databases. Following which, a regional survey was constructed and completed by HST and pleural consultants from five hospitals within the Peninsula deanery between April–July 2022. Data collected included participants demographics, attitudes, training, experience, and clinical knowledge. Outcomes were collated and comparisons made across groups using SPSS. A *p*-value of < 0.05 was defined as significant.

**Results:**

The scoping review returned six papers. Salient findings included low self-reported procedural confidence levels, poor interventional selection for patient cases, inadequate site selection for CTI and 1 paper reported only 25% of respondents able to achieve 5–10 CTI annually. However, all papers were limited by including grades other than HST in their responses.

The regional survey was completed by 87 HST (12 respiratory, 63 non-respiratory medical HST and 12 intensivists/anaesthetists HST). An additional seven questionnaires were completed by pleural consultants. Respiratory HSTs performed significantly more Seldinger CTI than general and ICM/anaesthetic registrars (*p* < 0.05). The percentage of HST able to achieve a self-imposed annual CTI number were 81.8, 12.9 and 41.7% respectively. Self-reported transthoracic ultrasound competence was 100, 8 and 58% respectively (*p* < 0.001). The approach to clinical management significantly differed with national guidance with pleural consultants showing an agreement of 89%, respiratory HST 75%, general HST 52% and ICM/anaesthetic HST 54% (*p* = 0.002).

**Conclusion:**

Compared to respiratory trainees, non-respiratory trainees perform lower numbers of Seldinger CTI, with lower confidence levels, limited knowledge, and a reduced perceived relevance of the skill set. This represents a significant training and service challenge, with notable patient safety implications.

**Supplementary Information:**

The online version contains supplementary material available at 10.1186/s12909-023-04978-8.

## Background

Pleural diseases may present at any time and require lifesaving intervention by competent practitioners. Traditionally chest tube insertion was performed by blunt dissection. However, with the emergence of the seldinger technique physicians now more commonly site narrow bore tubes for the management of non-traumatic pleural disease. Placement of wide bore drains by blunt dissection is often reserved for cases whereby the rapid removal of substances is required (e.g. traumatic haemothoraces/pneumothoraces) and is often performed by surgeons [1]. As such seldinger chest tube insertion (CTI) is a core capability of several higher speciality trainee (HST) curricula in secondary care. However, CTI is characterised as a high acuity low occurrence (HALO) procedure [2] and there are concerns regarding the degree of experience of generalists in pleural procedures [3]. Notably, in 2008, the National patient safety alert (NPSA) highlighted excessive complications and preventable deaths from CTI. Key concerns include poor patient selection, operator inexperience, unfamiliarity with equipment or national guidelines and a lack of appropriate supervision [4].

Subsequently, a number of developments to improve patient safety emerged, including the British Thoracic Society (BTS) 2010 guidelines on the use of bedside thoracic ultrasound (TUS) for pleural effusions [5]. Best practice tariffs and hospital bed pressures have encouraged the development of ambulatory pathways and have supported greater access to definitive procedures (thoracoscopy and indwelling pleural catheter insertion) [3, 6]. As such, pleural teams have developed to provide specialist services within working hours [7]. Such restructuring is likely to impact on the generalist physicians’ exposure and management of pleural disease [3].

This paper sets out to investigate the current practices of respiratory, general medical, and ICM/anaesthetic HST in performing Seldinger chest tube insertions in acute care settings in the Peninsula deanery.

## Objectives

We aimed to establish the current experiences, perceptions, and barriers of medical HST performing Seldinger chest tube insertions in UK hospitals, and to establish the attitudes, training, experience, and knowledge of general, respiratory, and ICM/anaesthetic HST in performing Seldinger chest tube insertions in acute care settings are in the Peninsula deanery.

## Methods

**Fig. 1:**
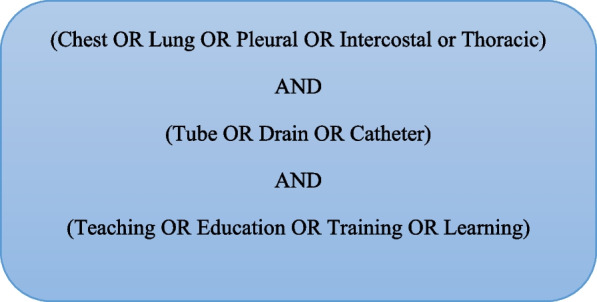
Literature review key terminology

**Fig. 2:**
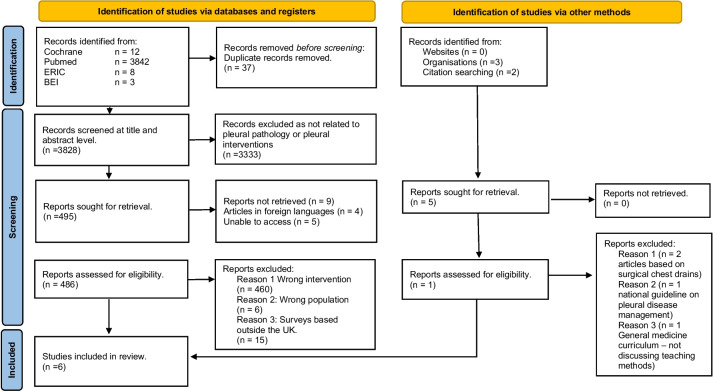
PRISMA 2020 flow diagram for new systematic reviews which included searches of databases, registers and other sources Modified from Page et al. [8]

A scoping review was performed (and subsequently updated on the 6/4/23) using a Boolean search with key words identified in Fig. 1. Cochrane, Pubmed via medline, ERIC and British education Index via EBSCO host databases were searched. Exclusion criteria were as follows: duplicate papers, papers not in English language, papers focusing on HST experience outside the UK and papers that did not include a HST experience (Specialist registrar trainee year 3 and above, “ST3+” or equivalent). A Preferred Reporting Items for Systematic Reviews and Meta-Analysis (PRISMA) flow chart is shown in Fig. 2 [8].

### Questionnaire development

Following the scoping review of previous HST surveys, a questionnaire was developed (Additional file 1: Appendix 1). This was subsequently tested with seven respiratory consultants with expertise in pleural disease (either being trust pleural leads or having advanced pleural interventional skills). Key survey areas included attitudes, training, experience, and clinical knowledge.

### Attitudes

Participants were asked a series of questions on their perception of the relevance and importance of being competent in siting a CTI and their confidence in performing the procedure. Answers were provided using a 5-point descriptive Likert scale from extremely important to irrelevant.

### Training and experience

Participants stated the numbers of CTI perceived to be required to attain and subsequently maintain independent competence**.** Participants were asked for their experience and confidence with CTI and TUS.

A still image of a septated pleural effusion was provided and participants were informed of the position of the ultrasound probe on said body and asked to identify three components (liver, diaphragm and septated effusion).

### Clinical knowledge

Four common case scenarios of presentations to the acute admissions unit were provided. Participants were asked to choose the best of five pre-specified answers. The correct answers were determined using BTS guidelines 2010 and independently assessed alongside responses by seven respiratory physicians with a subspecialty interest in pleural disease.

### Study participants

Higher speciality trainees (ST3+ or equivalent) across general medical services across five hospitals in the Peninsula deanery were invited to partake in a multicentre questionnaire between April 2022–July 2022. Participants were identified either from the acute medical rotas at each of the trusts or identified via the ICM/anaesthetic deanery wide mailing list. Participants were invited by email with an attached hyperlink to a google survey form. Non-responders were sent two further prompting emails. HST responses were then extracted and collated.

### Data analysis

Participant responses were entered on a Microsoft excel spreadsheet version 2019 and subsequently analysed with IBM® SPSS® version 28.0.0.0.0. Normality was tested using the Shapiro-Wilk test. Parametric and non-parametric data were described using mean (standard deviation) and median [interquartile range] respectively. Parametric comparisons were analysed using Analysis of Variance (ANOVA) and non-parametric comparisons with Kruskal-Wallis test. Proportional data was expressed as n(%) and compared using Chi-squared test. A *p*-value of < 0.05 was defined as significant. Participant number is provided for any answers which received an incomplete number of responses.

### Ethical approval

The study was approved as a multicentre service evaluation by the Plymouth University Hospitals NHS Trust reference number CA_2022–23-010.

## Results

### Scoping review

Six publications were identified that sought to address general medical HST experience of CTI in the UK [9–14]. Papers were published between 2005 and 2021. All papers were surveys, five were multicentred and three were restricted to their local postgraduate deanery. All surveys assessed participant responses using a Likert scale or equivalent. All papers included responses from core medical training or consultants in addition to HST.

The findings would suggest that despite the NPSA of 2008, a large proportion of medical trainees have no TUS training, [11, 12] select inappropriate patients to undergo pleural interventions, [11] and when they perform CTI for pneumothoraces select inappropriate sites [10, 14]. Guidelines and checklists appear not to be followed [13]. Furthermore, self-reported confidence amongst non-respiratory specialists is low [12, 13]. A major limitation of previous work is the amalgamation of responses from practitioners who do not hold responsibility for performing CTI. Table 1 provides a summary table of previous published surveys. The remainder of this article will focus on a multicentre service evaluation of HST.

**Table 1:** UK surveys involving Higher speciality trainee experience of CTI

Author, Year, Region	Population	Aim	Summary of pertinent results
Connick 2009 East Anglia [9]	181/186 hospital practitioners from 2 centres All grades responded including 55 registrars	To assess self-reported confidence in performing practical procedures.	58% of all respondents felt confident in CTI and 70% felt confident in pleural aspiration. 61% of Registrars/Consultants felt all practitioners should be competent in pleural aspiration and 56% in CTI.
Corcoran 2015 Oxford [11]	117 general physicians responded including 48 medical registrars and 38 core medical trainees	To ascertain doctors’ perception of the minimum numbers of procedures to attain/maintain CTI competence, experience to date of CTIs and assess knowledge	51% of respondents managed scenarios correctly. 89% registrars performed enough to achieve competence, 25% performed enough in last year to maintain competence (5–10 to achieve and 5–10 per year to maintain). 47.7% of trainees (core medical trainees and registrars) never had access to simulation training in CTI. 57% of trainees never had any TUS training and didn’t expect any in the future.
Corcoran, 2016 Oxford [10]	66 participants – 21 Core medical trainees and 45 registrars across 4 hospitals.	To identify whether medical trainees can identify a safe site in which to perform a CTI for a pneumothorax in the safety of triangle	45 registrars performed CTI previously and 20/21 core medical trainees had performed a CTI previously. 60.6% of all trainees could identify a site within the triangle of safety (there was no statistical significance between HST and core medical trainees *p* = 1.0)
Griffiths, 2005 Sheffield [14]	55 junior doctors (from foundation year 1 – registrars) surveyed in a single teaching hospital. Only 2 doctors were registrars	To assess whether junior doctors could safely identify an area inside the safe triangle to site a chest drain	45% of junior doctors stated they would site a chest drain outside the safe triangle. 28/55 junior doctors had performed a chest drain. 3/11 who had performed a chest drain under supervision intended to site a chest drain outside the safe triangle and 3/17 who had performed a chest drain unsupervised would site this outside the safe triangle
Lagan 2015 Wirral [12]	156/269 doctors returned survey. 73 registrars and 83 core trainees	To assess trainee procedural confidence. To explore procedural training and exposure to procedures To demonstrate correlation between confidence and exposure.	70% wanted to perform more procedures, 3.8% wanted fewer procedures and 26% wanted the same number. 80% disagreed that “practical procedures should be reserved for specialists with an interest in them”. 98.7% agreed they should be procedurally competent in case of emergencies. 21% registrars not independent in chest drains and 61% core medical trainees not independent. 81% not independent in TUS for pleural procedures and 40% had no training in chest ultrasound for pleural procedures. Procedural exposure statistically associated with confidence (*p* < 0.003) and significant correlation between chest ultrasound and confidence for pleural procedures (*P* < 0.017)
Miller, 2020 Torbay (Poster presentation at national conference) [13]	137 responses from 8 hospitals including 39 general medical registrars	Aims were to identify pleural practices in the region including the number of procedures performed, self-rated confidence of operators undertaking them out of hours and the availability of standard operating procedures (SOPs), safety checklists and procedure rooms	90.4% of respondents said out of hours procedures were the responsibility of the general medical registrars. 39 General medical registrars had a mean confidence of 2.4/5 (95% CI of 2.09, 2.78) in performing emergency CTI. Respiratory registrars had an average confidence of 3.9/5 (95% CI 3.26,4.55). 70.7% of general medical registrars desired further training. 53.3% of respondents knew of a pleural safety checklist and only 20.7% were using this regularly. 53% did not know where to find a standard operating procedure for pleural procedures. 18% of respiratory trainees had regular access to a procedural room.

### Survey results

#### Participants and demographics

A total of 87/116 HST completed the survey, including 12/14 respiratory HST, 63/73 general HST and 12/29 ICM/anaesthetic HST. There were 50/87 (57.5%) male respondents. Seniority included 25/87 (28.7%) at ST3/IMT3 level, 35/87 (40.2%) at ST4/5 level and 27/87 (31.0%) at ST6/ST7 level.

#### Attitudes

All respiratory HST reported CTI as being “extremely” or “very important,” dropping significantly to 30/63 (48%) for general HST and 8/12 (67%) for ICM/anaesthetist HST (*p* < 0.001). Respiratory HST were more confident in unsupervised CTI, than general HST and ICM/anaesthetic HST; 11/12 (92%) versus 10/63 (16%) versus 9/12 (75%) respectively (*p* < 0.001).

#### Experience

Annually respiratory trainees performed a median of 7.8 [IQR 3.8–15] CTI. This was significantly higher than for both general HST 0 [IQR 0–1] and ICM/anaesthetic HST 1 [IQR 0–2] (*p* < 0.001). The perceived numbers of CTI to attain competence across respiratory, general and ICM/anaesthetist HSTs were 8.8 [IQR 5.3–10], 5 [IQR 3–10] and 8.75 [IQR 5–10]. The perceived annual number of procedures to retain CTI competence was similar across groups with a mean of 3–4 per year. Pleural consultants perceived similar numbers of CTI necessary to achieve and retain competence as trainees. (M = 7.5 [IQR 5–10], *p* = 0.242 and M = 4.0 [IQR 2.5–5], *p* = 0.733). However, the proportion of HST achieving a self-imposed number to retain competence differed amongst cohorts, with targets reached by 9/11(81.8%) of respiratory HST, 8/62 (12.9%) of general HST and 5/12 (41.7%) of intensivists/anaesthetists.

#### Training

Only 8% of respiratory HST, 40% of general HST and 50% of ICM/anaesthetics HST received some form of pleural teaching in the preceding 6 months. The most common mode of teaching for general HST was the use of mannequins (25%) and bedside teaching for ICM/Anaesthetists (33%).

#### TUS training

Self-reported TUS competence differed across trainees, with 12/12 (100%) respiratory HST, 5/63 (8%) general HST and 7/12 (58%) of ICM/anaesthetists reporting TUS competence (*p* < 0.001). While all respiratory HST had TUS accredited with a national award body, only 3% of general HST and 33% of ICM/anaesthetic HST had achieved this.

#### TUS image interpretation

**Fig. 3:**
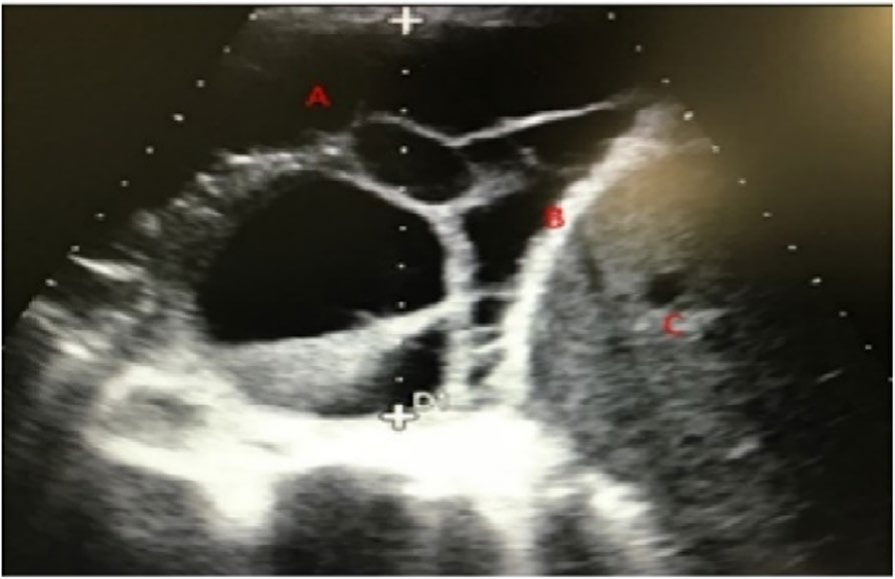
Displaying a transthoracic ultrasonographic image demonstrating a septated pleural effusion

Participants were invited to identify three important ultrasonographic findings as seen in Fig. 3.

All pleural consultants correctly identified a septated pleural effusion, the liver and diaphragm. Trainees correctly identified all features in 11/12 (91.7%) of respiratory HST, 29/63(46%) of general HST and 10/12(83.3%) of ICM/anaesthetic HST. While all respiratory and ICM HSTs correctly identified the liver and diaphragm, only 40/63 (63.5%) general HST were able to. For the general HST the commonest misinterpretation was of the septated effusion representing lung.

#### Clinical knowledge

Following the clinical scenarios, participant responses were in line with the BTS 2010 guidance in 25/28(89.2%) pleural consultants and 36/48 (75.0%) respiratory HST [5]. This dropped significantly to 148/252(58.7%) general HST and 26/48 (54.2%) of ICM/anaesthetic HST (*p* < 0.01). Where intervention for pneumothoraces was indicated, there was a trend towards using TUS for site selection with 14.3% of pleural consultants, 42.0% of respiratory HST, 58.7% of general HST and 66.7% of ICM/anaesthetic HST selecting this option. A comprehensive overview of procedural selection is provided in Table 2.

**Table 2:** Single best answer selections for each of the four clinical vignettes

Clinical Vignette	Single Best Answer (SBA)	Pleural consultant (participants n/N (%))	Respiratory HST (participants n/N (%))	General HST (participants n/N (%))	ICM/anaesthetic HST (participants n/N (%))
A 32 year old male with no past medical history presents with left sided chest pain. He is not breathless. A chest X-ray shows a left pneumothorax measuring 3 cm at the apex and 1.5 cm at the hilum. Oxygen saturations 95% on room air. Your treatment plan is to:	A Discharge home, outpatient follow up^a^	6/7 (85.7)	11/12 (91.7)	45/63 (71.4)	6/12 (50.0)
	B Pleural aspiration without the use of ultrasound	1/7 (14.3)	0	4/63 (6.3)	1/12 (8.3)
	C Pleural aspiration with the use of ultrasound	0	1/12 (8.3)	12/63 (19.0)	3/12 (25.0)
	D Chest drain without the use of ultrasound	0	0	1/63 (1.6)	1/12 (8.3)
	E Chest drain guided with the use of ultrasound	0	0	1/63 (1.6)	1/12 (8.3)
A 57-year-old male with COPD presents with breathless. A CXR shows a right pneumothorax measuring 3 cm at the hilum. Your treatment plan is:	A Discharge home, outpatient follow up	0	0	0	0
	B Pleural aspiration without the use of ultrasound	0	1/12 (8.3)	0	0
	C Pleural aspiration with the use of ultrasound	0	0	10/63 (15.9)	0
	D Chest drain without the use of ultrasound^a^	6/7 (85.7)	6/12 (50.0)	16/63 (25.4)	4/12 (33.3)
	E Chest drain guided with the use of ultrasound	1/7 (14.3)	5/12 (42.0)	37/63 (58.7)	8/12 (66.7)
A 50-year-old female with known lung cancer presents with shortness of breath. A Chest X-ray reveals a large right sided pleural effusion not present on a CXR performed four months earlier. Your treatment plan is:	A Discharge home, outpatient follow up	0	0	2/63 (3.2)	0
	B Pleural aspiration without the use of ultrasound	0	0	1/63 (1.6)	0
	C Pleural aspiration with the use of ultrasound^a^	7/7 (100)	11/12 (91.7)	42/63 (66.7)	8/12 (66.7)
	D Chest drain without the use of ultrasound	0	0	0	0
	E Chest drain guided with the use of ultrasound	0	1/12 (8.3)	18/63 (29)	4/12 (33.3)
A 65-year female with known lung cancer presents with dyspnoea on exertion. She previously had 1.5 L of fluid removed from the right pleural space 2 weeks ago, cytology demonstrated adenocarcinoma cells present. Exercise tolerance < 10 yards. Oxygen saturations 90% on air. CXR shows a large right sided pleural effusion. Your treatment plan is:	A Discharge home, outpatient follow up	0	0	1/63 (1.6)	2/12 (16.7)
	B Pleural aspiration without the use of ultrasound	0	0	0	0
	C Pleural aspiration with the use of ultrasound	1/7 (14.3)	4/12 (33.3)	17/63 (27.0)	2/12 (16.7)
	D Chest drain without the use of ultrasound	0	0	0	0
	E Chest drain guided with the use of ultrasound^a^	6/7 (85.7)	8/12 (66.7)	45/63 (71.4)	8/12 (66.7)

^a^Single Best Answer as per British Thoracic Society National guidelines. n/N, where n = number of responses selecting this option, *N* = total number of participants in this subgroup. Please note the clinical vignettes are representative of commonly occurring cases presented to the unselected medical take and do not contain any patient data

#### Improving procedural safety

**Fig. 4:**
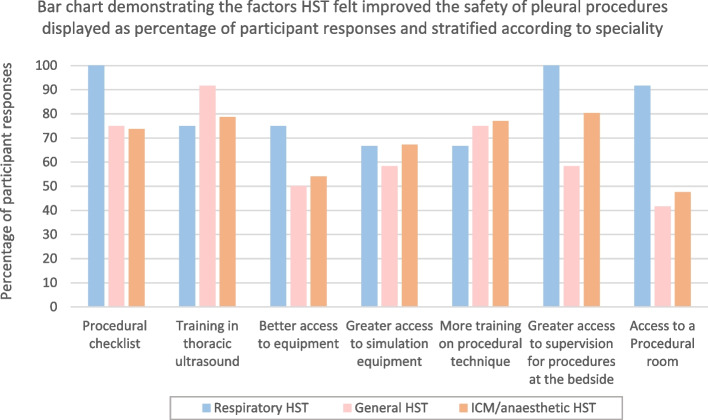
Demonstrating the factors higher speciality trainees felt would improve the safety of pleural procedures

The majority of HST stated that procedural checklists, access to a procedural room, better access to equipment and enhanced training opportunities would enhance patient safety. Training opportunities included access to simulation equipment, training on procedural technique, greater supervision for bedside procedures and training in TUS (Fig. 4) Free text responses to improve patient safety included standardising the equipment across the deanery, restricting the performance of CTI to specific specialities, and providing appropriate regular training opportunities to retain competence.

## Discussion

This study has demonstrated the majority of general HST are not receiving sufficient practice to achieve a self-imposed number of CTI to retain clinical competence. Lack of exposure to CTI coincides with a reduction in confidence in being able to confidently perform a Seldinger CTI and a reduction in the relative importance non-respiratory HST place on being able to perform CTI independently. Moreover, non-specialists appear to deviate significantly from both specialists and the application of the BTS guidelines in managing pleural disease. The findings of this study are supported by similar previous studies. In 2015, Corcoran et al demonstrated only 25% of general HST were able to meet a self-imposed minimum standard of 5–10 CTI/year to retain said skills [11]. However, this study has found further reduction with only 12.9% of generalists meeting an individualised minimum standard, despite their expectations for annual numbers to retain competence being less. We observed an apparent shift in attitude away from the relevance of being CTI competent. In 2015 Lagan polled core medical and HST and found 98.7% of respondents felt they should be “procedurally competent in case of emergency” [12]. However, in this study only 48% of generalists and 67% of ICM/anaesthetic HST stated it was “extremely” or “very important” for them to be procedurally competent in CTI.

In terms of TUS, the BTS guidelines mandate bedside TUS being performed prior to CTI for pleural effusions [5]. Despite this the majority of generalists in our study were not adequately TUS trained. Furthermore, the majority of non-respiratory specialists have no formal national accreditation despite the BTS having streamlined TUS training with the publication of a training standards and accreditation framework [15]. Given the BTS 2010 guidelines strongly emphasise bedside TUS guidance for CTI site selection for effusions, without TUS training, opportunities for generalists to perform CTI are limited. Moreover, Lagan documented procedural confidence was significantly associated with both exposure and TUS competence [12]. TUS is a core capability for respiratory trainees [16] and TUS training may partially explain the enhanced confidence rates found in respiratory HST when compared to non-respiratory trainees [13]. As such, future research may wish to consider the effects of incorporating point of care ultrasound into the 2022 acute medical curriculum on operator procedural confidence [17]. Notably although TUS has become widespread, current practice has deviated away from the BTS 2010 guidelines. Polled specialists are also favouring TUS for site selection of pneumothoraces. In this context TUS may be used to confirm a pneumothorax, or to confirm various site selection safety parameters (supra-diaphragmatic position, exclude proximity of visceral organs or to exclude aberrant blood vessels). This may indicate a two-tier system of safety for patients undergoing pleural intervention.

Although seldinger CTI for non-traumatic pleural disease has historically been performed by general medical HST, the lack of exposure and training of non-respiratory HST in pleural procedures and pleural disease management represents significant concerns [12]. In spite of which, medical registrars are the medical emergency team leaders, are often the most senior out of hours on-site doctor on the medical team and trusts rely upon this cohort to be perform emergency pleural procedures. As such CTI has remained a key capability on the general medical curriculum [18]. However, providing adequate training for generalists is challenging. Pleural disease has increasingly become a respiratory sub-specialism. Many trusts have or are in the process of establishing pleural teams to manage cases within hours. Such teams consist of a mixture of professionals, which may include non-physician specialities and this will impact on the opportunities for generalists to obtain experience in pleural disease [3]. Furthermore, an expanding repertoire of available procedures (ambulatory drains for pneumothoraces, indwelling pleural catheters and medical thoracoscopy for effusions) means different options exist for managing non-life-threatening pleural conditions. Such options will depend on local provisions, but such choice will impact on the numbers of patients undergoing seldinger CTI. Indeed, reflective of current practice there is discrepancy amongst the seven pleural consultants polled in this survey and this variation may be due to the skill mix and available provisions at each trust.

Moreover, the clinical benchmark used for the clinical vignettes was the 2010 BTS guidelines [5]. Specialists may be aware of advances in pleural disease management and the authors acknowledge subsequent to this survey updated BTS guidelines have been released. The BTS 2023 guidelines acknowledge a range of options exist for non-life-threatening pleural disease with a focus on patient choice, a move towards more conservative or ambulatory management of primary spontaneous pneumothoraces and more rapid definitive treatment of suspected malignant pleural effusions. Whilst the single best answers still apply, the authors acknowledge alternative strategies exist for managing rapidly recurring malignant pleural effusions such as indwelling pleural catheters, talc pouldrage or surgical pleurodesis in selected cases [19]. Despite which, the generalists’ choices in this study deviate significantly from the pleural consultants, respiratory HST and the BTS guidelines. This would indicate a gap in knowledge (or application) that needs to be addressed in order to standardise pleural disease management.

A strong dichotomy in the performance of seldinger CTI currently exists. Patients who present outside of working hours are being managed by non-respiratory specialists with little knowledge or exposure to pleural disease management. Whereas those who are highlighted within hours are managed by increasingly subspecialist teams. Indeed, expert opinions have suggested restricting performance of pleural procedures to subspecialists [3, 20] and therefore consideration of pleural team extension to cover out of hours would be warranted.

Alternatively, significant investment in training generalists could be considered. Given the infrequency of HALO procedures such as CTI, implementation of comprehensive simulation based procedural training curricula may support the traditional learning opportunities [21, 22]. Indeed, HST perceived training improvements to be key to improving patient safety. These include TUS training, greater access to simulation equipment, further training on CTI technique and greater availability of bedside procedural teaching.

Either suggestion comes with considerable economic and logistic implications. However, maintaining the status quo has significant patient safety implications. If non-specialists HST are expected to perform pleural interventions out of hours, then there needs to be sufficient training and exposure to ensure they are competent.

### Limitations

This paper focused on Seldinger CTI and did not poll surgical/blunt dissection CTI which could be used as an alternative method to site CTI (albeit normally restricted to cases of trauma or post-surgical interventions). The scope of the work did not include non-medical specialties, but further work should be done to address similar themes across surgical and emergency medicine HSTs. Although responder bias and restriction to a single deanery are limitations, our findings are consistent with similar studies and the results cannot be ignored.

Lastly, although patient safety concerns are highlighted, no attempt to benchmark competence was performed. By definition HALO procedures are infrequent and extensive resources would be required to demonstrate harm from any particular cohort. Likewise, the parameters to determine harm would need careful consideration. Complication rate in itself is of limited value as specialists may be referred more complex procedures and perform procedures despite relative contraindications (coagulopathy, tethered lung etc) which incurs greater risk.

## Conclusion

This study highlights the current training and patient safety challenges in providing comprehensive pleural interventional service delivery on a deanery-wide level. Reorganisation of pleural interventional services with a greater focus on ambulatory care has revolutionised patient care. Likewise, improvements in patient selection, streamlining of pleural pathways and the widespread adoption of point of care thoracic ultrasound for effusions will affect the number of unnecessary procedures performed and avoidable complications incurred.

However, opportunities for non-respiratory higher speciality trainees to perform Seldinger chest tube insertion have dwindled and few are able to achieve self-imposed minimum standards to retain CTI as a core capability. Moreover, few non-respiratory HST are trained in thoracic ultrasound which is mandated prior to procedures for pleural effusions, and few are attending regular CTI training to ensure sufficient skill retention. As such the relevance of the skill set for non-respiratory HST has diminished, as has the operator confidence in performing CTI. Alongside which, non-respiratory HST are significantly deviating in procedural selection from their respiratory counterparts and national guidelines.

To address these training and patient safety concerns, significant investment is required to ensure comprehensive delivery of pleural procedures by competent practitioners. Either in the form of extensive training programs if non-specialists are to continue to provide this care, or in ensuring adequate provisions are available to staff a dedicated 24/7 pleural service delivery.

### Supplementary Information

**Additional file 1: Appendix 1.** Pleural procedure Questionnaire administered to higher speciality trainees.

## Data Availability

The datasets used and/or analysed during the current study are available from the corresponding author on reasonable request.

## Abbreviations

BTS: British Thoracic Society
CTI: Chest Tube Insertion
HALO: High acuity Low Occurrence
HST: Higher Speciality Trainee
IMT: Internal Medicine Trainee
ICM: Intensive Care Medicine
NPSA: National Patient Safety Alert
ST: Speciality Trainee
TUS: Transthoracic Ultrasound
UK: United Kingdom
