# Supplementary material for: Training, experience, and perceptions of chest tube insertion by higher speciality trainees: implications for training, patient safety, and service delivery
Source: BMC Med Educ. 2024 Jan 3;24:12. doi: 10.1186/s12909-023-04978-8 (PMC10765639; doi:10.1186/s12909-023-04978-8)
Supplement: Supplementary file 1 — Additional file 1: Appendix 1. Pleural procedure Questionnaire administered to higher speciality trainees. [file 12909_2023_4978_MOESM1_ESM.docx]

*Appendix 1 - Pleural procedure Questionnaire administered to higher speciality trainees*

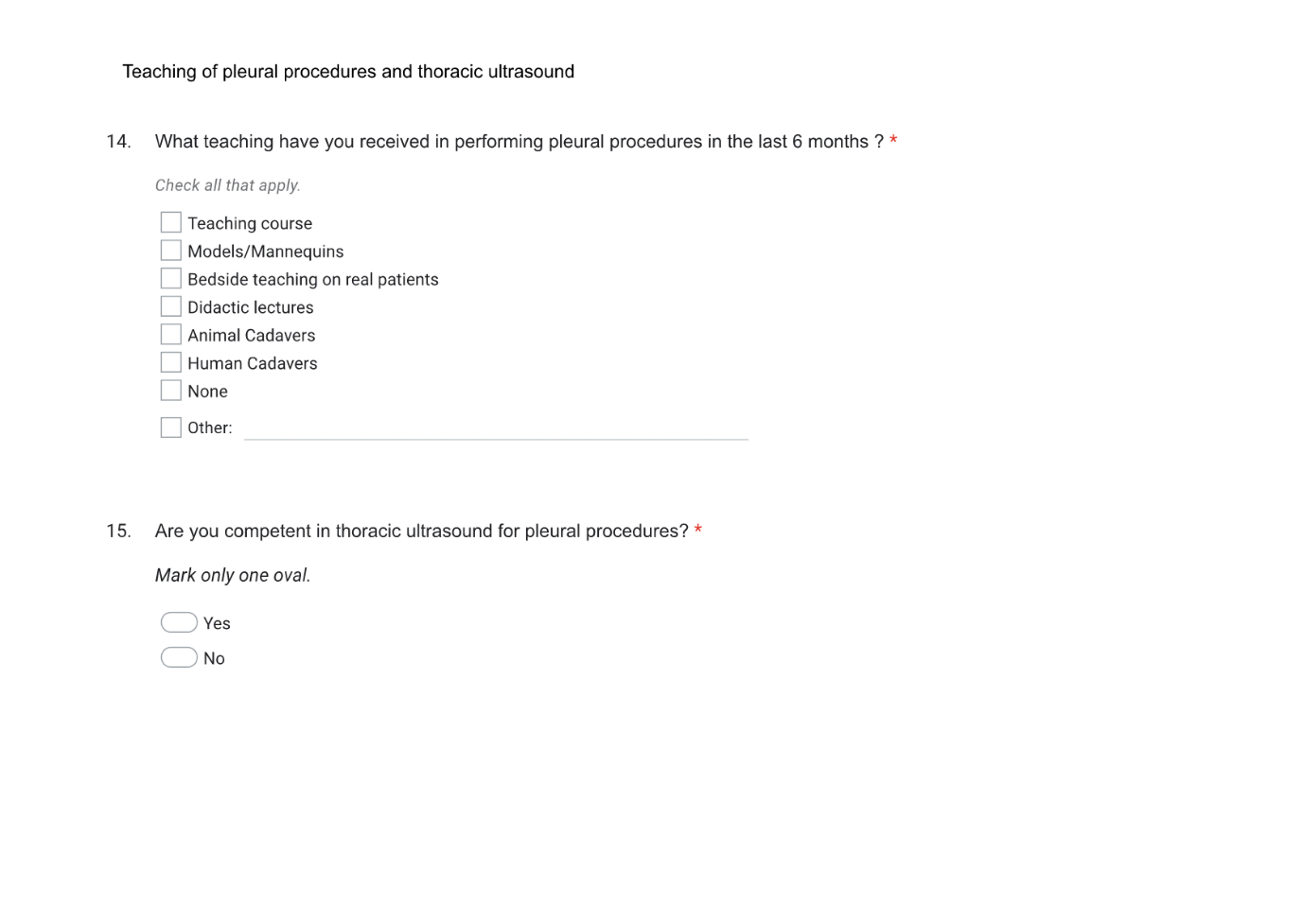

19. For respiratory trainee's,  please select what level of experience best describes your exposure of the following advanced interventional procedures during your training to date.

*Check all that apply*

|  | No experience | Skills laboratory experience | Observations of procedures on patients | Some experience under direct supervision | Extensive experience, entrusted to act unsupervised and seeks help when required | Fully competent to act independently and manage complications unsupervised |  |
| --- | --- | --- | --- | --- | --- | --- | --- |
| Indwelling pleural catheter |  |  |  |  |  |  |  |
| Lymph node assessment and Fine needle aspiration |  |  |  |  |  |  |  |
| Medical thoracoscopy under Local anaesthesia |  |  |  |  |  |  |  |
| Ultrasound guided pleural or lung biopsies |  |  |  |  |  |  |  |
| Ultrasound guided real time pleural aspiration/chest drain placement |  |  |  |  |  |  |  |
| Surgical chest drains |  |  |  |  |  |  |  |
